# Use of Complementary and Alternative Medicine Among Patients With Epilepsy and Diabetes Mellitus, Focusing on the Outcome of Treatment

**DOI:** 10.3389/fnins.2021.787512

**Published:** 2022-01-11

**Authors:** Michael Magdy Fahmy Girgis, Klára Fekete, Nóra Homoródi, Sándor Márton, István Fekete, László Horváth

**Affiliations:** ^1^Department of Pharmaceutical Surveillance and Economics, Faculty of Pharmacy, University of Debrecen, Debrecen, Hungary; ^2^Department of Neurology, Faculty of Medicine, University of Debrecen, Debrecen, Hungary; ^3^Institute of Cardiology, Faculty of Medicine, University of Debrecen, Debrecen, Hungary; ^4^Institute of Political Science and Sociology, Faculty of Arts, University of Debrecen, Debrecen, Hungary

**Keywords:** complementary and alternative medicines, epilepsy, diabetes mellitus, outcome, adherence

## Abstract

**Introduction:** Millions all over the world live with epilepsy, and they may require long-term drug treatment. The use and interest in complementary and alternative medicine (CAM) have grown over the previous years. Coadministration of herbal products with medicines may result in adverse drug reactions (ADRs) and/or unfavorable interactions. The aims of this study were to determine the prevalence of CAM use among patients with epilepsy, to compare the results to those of the patients with diabetes mellitus (DM), to reveal factors that may drive the use of CAM, and to measure outcomes and adherence. It was also our intent to have state-of-the-art information on CAM use in our region among patients with the two diseases above.

**Materials and Methods:** We conducted a non-interventional study using a self-developed questionnaire. It was distributed among adult patients with either epilepsy or DM who also suffered from cardiovascular consequences. A database was compiled from the anonymous questionnaires filled in voluntarily by the patients. Basic statistics were used to analyze this database.

**Results:** A total of 227 questionnaires were filled in by 127 patients (55.9%) with epilepsy and 100 patients (44.1%) with DM. Mean age was 54.54 ± 17.33 years. Of the patients, 50.2% were male. Average body weight was 80.3 ± 17.3 kg. Of the patients, 22 (9.7%) used CAM because they believed in CAM. Two of them reported ADRs. Among the patients with epilepsy, the ratio was only 7.9% compared to 12% among those with DM. While the number of CAM users was higher among younger patients with epilepsy, it was the elderly patients with DM who tended to use CAM.

**Conclusion:** Attention should be paid to reliance on CAM during the follow-up. Our finding that health-conscious patients tend to use CAM more often (than the general population) may indicate it is necessary to discuss CAM usage sincerely. CAMs modulating cytochrome P450 (CYP) enzymes were the most common, leading to interactions with medication used and resulting in ADRs. This shows the importance of educating patients and treating team including clinical pharmacists in this field.

## Introduction

### Some Challenges of the Treatment of Patients Living With Epilepsy and Diabetes Mellitus

Millions all over the world suffer from *epilepsy*. It affects both sexes and all ages with slightly higher prevalence and incidence in men. There are dozens of risk factors closely associated with the onset of epilepsy (Walsh et al., 2017). Greater frequency of stroke, neurodegenerative diseases, and tumors are the most common etiological factors among the elderly (Beghi, 2020). In most cases, epilepsy requires lifelong treatment and/or follow-up in different life stages, posing a challenge for the patient, family, and attending epileptologist alike.

Despite patients having access to a high variety of new antiepileptic drugs (AEDs) in many parts of the world, the old ones are still essential for different reasons (Vajda and Eadie, 2014).

*Diabetes mellitus* (*DM*) is a burden of health care, and it leads to several complications, e.g., macrovascular complications (Amutha and Mohan, 2016). DM also requires lifelong treatment with the importance of lifestyle changes.

In a population-based study of epilepsy incidence in association with type 2 diabetes mellitus (T2DM) and severe hypoglycemia based on Taiwan’s National Health insurance claims incorporating 751,792 people with T2DM and 824,253 matched controls identified in 2002–2003 with follow-up until incidence of epilepsy in 2011 (Chin-Li et al., 2018), it was found that T2DM may increase the risk of epilepsy independently of severe hypoglycemia. That study showed 50% elevated hazard of epilepsy in T2DM patients consistently observed for all gender and age stratifications. Moreover, both epilepsy and T2DM patients are commonly prescribed many medicines simultaneously for long periods, and they may seek complementary and alternative medicine (CAM) therapies, which makes it useful to compare patients’ adherence to their prescribed medications, CAM use, and the impact on patients’ satisfaction.

Both diseases are common and have standardized treatment protocols. These diseases have only one pharmacological class as a treatment group; on the contrary, hypertension has more. Similar to DM, epilepsy rarely has severe consequences such as malignant arrhythmia, prolongation of QT, and sudden unexpected death in epilepsy (SUDEP) (Devinsky et al., 2016).

### Complementary and Alternative Medicine

According to the National Center for Complementary and Alternative Medicine (NCCAM) and National Institutes of Health (NIH), “complementary and alternative medicine or modalities (CAM)” was defined as health care approaches with a history of use or origins outside of mainstream medicine (Kramlich, 2014). The use and interest in CAM have grown over the previous years, which emphasizes the importance of considering various contributing factors. Studies have reported a significant increase in CAM use among patients living with chronic diseases (Bücker et al., 2008). The main motivation for CAM use is to avoid polytherapy and disappointment due to the results after conventional therapies. Media, family, and friends are the main sources of information for CAM. Among the elderly, increasing CAM use could be detected, including using herbal supplements concomitantly with conventional medications. This might be due to various concerns like polypharmacy, decreased organ functions, and increased sensitivity to some medications (Elmer et al., 2007). Patients tend to use CAM thinking that CAM therapy is safe and being unaware of the potential adverse effects. Unfortunately, these expose them to an increased risk for admission to an intensive care unit (Bello et al., 2012). CAM therapies may be considered as a choice when conventional care turns out to be unsuccessful or when therapy escalation is deemed to be too risky or no longer beneficial for severely ill patients (Gilmour et al., 2011).

### Importance of Complementary and Alternative Medicine in Epilepsy and Diabetes Mellitus

Complementary and alternative medicine therapies, especially herbal medication, can modify the effect of AEDs and antidiabetics, even decrease them and result in ineffectiveness; moreover, they can cause toxic adverse drug reactions (ADRs). CAM may have unfavorable interactions with antidiabetics. Herbals, as an example of CAM, can affect clinical safety and efficacy *via* additive/synergistic or antagonistic interactions among the herbal components and drug molecules. While negative or harmful interactions tend to receive more attention due to safety considerations, additive/synergistic effects induced by herbal drug interactions may result in an enhancement of desired pharmacological effects (Gupta et al., 2017).

*Gymnema sylvestre* is an example of an unfavorable herbal interaction with antidiabetics. In an animal study using a chemically induced diabetic rat model, a decrease in plasma metformin concentration and increase in blood glucose levels were seen in animals treated with the combination of gymnema tea and metformin when compared to those receiving metformin alone, suggesting an antagonistic interaction between metformin and gymnema (Raja et al., 2013).

Traditional medicines for the treatment of DM are probably based mainly on treatment of its obvious symptoms of pronounced thirst and polyuria (Marles and Farnsworth, 1995). Nature is an outstanding source of antidiabetic medicines (Chang et al., 2013), and plants are valuable dietary supplements to improve blood glucose control and prevent long-term complications in T2DM (Gallagher et al., 2013). Folklore remedies are used for managing DM due to their non-toxic nature, cost-effectiveness, easy availability, and long-lasting effects over their synthetic counterparts (Bhagour et al., 2016). Certain fruits and vegetables are functional foods, and their consumption reduces the incidence of T2DM. Hypoglycemic effects of fruits and vegetables may be due to their inducing nature on pancreatic β-cells for insulin secretion or bioactive compounds such as flavonoids, alkaloids, and anthocyanins, which act as insulin-like molecules or insulin secretagogues (Beidokhti and Jager, 2017). Examples of used antidiabetic CAM therapies are avocado, *Musa paradisiaca*, *Aloe vera*, barberry, caraway, olive oil, and dill.

Focusing on patients living with epilepsy in countries that apply a western-based medical system, CAM is used not only to enhance general health but also to prevent seizures or to alleviate symptoms of comorbidities or the ADRs of antiepileptic medications (Ekstein and Schachter, 2010). The most frequently taken products include ginseng, *Ginkgo biloba*, St. John’s wort, echinacea, garlic, and soy. Interestingly, the aforementioned products have not been reported to have either beneficial or detrimental effects on seizures, though their presumed activity on the P450 system could potentially lead to interactions with AEDs metabolized by the liver. Melatonin, kava kava, and valerian were reported to have sleep-inducing and anticonvulsant effects; however, melatonin and kava kava were also associated with the aggravation of epilepsy. Ephedra and caffeine have been linked to proconvulsive effects. Some infrequently used products have shown beneficial effects on seizures, epilepsy comorbidities, or complications of epilepsy including skullcap, grapefruit juice, hops, and omega-3 fatty acids. Other countries have widely practiced forms of traditional medicine, among which Ayurveda and traditional Chinese medicine (TCM) are the best known. In Ayurveda, epileptic patients are prescribed mixtures of natural products, containing herbal extracts (like *Acacia arabica*, *Acoruscalamus*, and *Bacoppamonnieri*), as well as animal ghee, honey, and milk. Likewise, TCM involves mixtures of different herbal extracts (each containing many active compounds) either to treat the seizure disorder directly or to maintain the general wellbeing of a patient.

### Possible Effects of Complementary and Alternative Medicine on Pharmacological Treatment

In many instances, herbal drugs are used simultaneously with modern drugs. Generally, all drugs with a narrow therapeutic index may either have increased adverse effects or be less effective when used in conjunction with herbal products (Mehmood et al., 2019). Efficacy and clinical safety can be affected *via* additive/synergistic or antagonistic interactions among the herbal components and drug molecules (Gupta et al., 2017).

Diabetes mellitus is one of the chronic diseases reported to increase the probability of CAM use (Bishop and Lewith, 2010). Many interactions between CAM and AEDs through different mechanisms like intrinsic proconvulsant properties have their effect on the cytochrome P450 (CYP) enzymes and P-glycoproteins or changing disposition of AEDs (Samuels et al., 2008). Besides the important ADRs caused by hepatic enzymes, it is very important to mention the role of P-glycoprotein in therapy-refractory epilepsies. It is a well-known fact that some AEDs may induce P-glycoproteins. These interactions are difficult to predict; moreover, most patients do not inform their physicians that they are taking such medicine.

The main goals of this study were as follows: to determine the prevalence of CAM use among our patients with epilepsy and to compare the results to those of the patients with DM, to reveal factors that may drive the use of CAM, and to measure the outcome and adherence in order to improve care. We were also interested whether there was any difference between the prevalence of CAM use in two different chronic disease groups, i.e., epilepsy and DM. Since in our region CAM prevalence had not been studied yet, our intent was to have state-of-the-art information about CAM use.

## Materials and Methods

### Patients

Two self-developed questionnaires (including open-ended and closed-ended questions) were used in order to investigate the use of CAM and outcomes among adult patients with epilepsy (127 patients) and DM (100 patients). In both groups, the patients were diagnosed at least 1 year prior to filling in the questionnaire. Patients previously diagnosed with epilepsy at the Department of Neurology by two senior epileptologists (IF and KF) took part in regular checkups in a tertiary university hospital. Patients with DM were treated at the departments of cardiology and neurology and already had vascular consequences (e.g., stroke or myocardial infarction) of the disease. As a result, they were hospitalized in a tertiary university hospital and had undergone percutaneous coronary intervention (angioplasty with stent or thrombolysis). Similarly to epilepsy, DM may require lifelong treatment and attention from the family and attending physician in different stages of life. Nevertheless, social acceptance is better in DM; it is not a stigmatizing disease, that is why this population was chosen for comparison.

The following inclusion criteria were used in *both groups*: (a) only adult patients were involved in this study and (b) patient willingness to participate on a voluntary basis.

Inclusion criteria in the *epilepsy group*: patient was diagnosed with epilepsy according to International League Against Epilepsy (ILAE) classification (Fisher et al., 2014) and took part in regular checkups prior to be involved in the survey.

Inclusion criteria in the *diabetes mellitus group*: patients with a primary diagnosis of DM and subsequent vascular consequences; and during the study period: the patient was hospitalized to treat a vascular event.

Exclusion criteria were as follows: (a) dependent patient, (b) serious medical condition, and (c) other major comorbidities.

### Questionnaire

Patients filled in the questionnaire anonymously and voluntarily between December 2018 and September 2019. It contained questions that were about patients’ demographics, lifestyle activities, seizure freedom, prescribed AEDs, adherence, satisfaction, reported adverse effects, CAM therapy, and quality of life. A patient was a “smoker” if he smoked actively, a “non-smoker” if he had never smoked, and “stopped smoking” if he quitted the habit at least 1 year before filling in the questionnaire. The self-developed questionnaire for DM sufferers had some questions identical with the one used in the other study on epileptic patients. In addition, patients living with DM had specific questions concerning diabetic diet, owning a glucometer at home, frequency of measuring blood glucose levels, family history of DM, last measured fasting blood glucose, last measured HbA_1c_%, prescribed antidiabetics, adherence, and general satisfaction with therapy.

Both aforementioned surveys were developed through the authors’ collaborative work. Surveys were reviewed and discussed by many clinicians and experts to obtain face validation.

Data were entered in a database for further evaluation. In order to reduce the risk of error, data were first entered into spreadsheet by one of the authors; later, two authors collaborated in reviewing the merged database.

### Outcome

Controlled disease as a good outcome was considered in case of patients living with epilepsy if they declared themselves as seizure-free, in case of patients with DM if fasting glucose level was <7.0 mmol/L and/or HbA_1c_ < 6.5%.

Adherence to prescribed medication was classified into three categories:

1 – Good (as long as patients had taken at least 90% of their prescribed medicines or maximum 3 days of drug holiday a month).
2 – Less often (as long as they had taken at least 50%–90% of their prescribed medicines).
3 – Poor (as long as they had taken at least < 50% of their prescribed medicines).

Physical activity was defined as any physical activity lasting at least 30 min a day.

The five-point Likert scale was used to measure a patient’s overall quality of life, where one meant wellbeing.

### Statistics

Statistical analysis was carried out using the SPSS for Windows 19.0 (SPSS Inc., Chicago, IL, United States) and Microsoft Office Excel 2016.

Two-sample *T* test and *F* test were used to analyze our patients’ data. Categorical variables were assessed using Pearson χ^2^ test and Fisher’s exact test.

As per standard pharmacovigilance practices, the values of the odds ratio (OR) were computed using 2 × 2 contingency table.

Significant differences were considered if *p* < 0.05.

## Results

Two hundred twenty-seven patients completed the questionnaire (127 patients with epilepsy and 100 patients with DM). Of them, 114 (50.2%) were male and 112 (49.3%) were female, while a participant did not state gender preference. Mean age was 54.54 ± 17.33 years; people with epilepsy were significantly younger.

Patients with *epilepsy* listed the following CAM used parallel to the anticonvulsant therapy: lemon balm (*Melissa officinalis*) (three patients), pomegranate (*Punica granatum*), valerian (*Valeriana officinalis*), rose hip (*Rosa canina*), aloe vera, Sedacur [contains lemon balm, valerian, hop (*Humulus lupulus*)], linden (*Tilia*), mint (*Mentha*), thymus, and senna.

Patients with DM reported the following CAM used parallel to the antidiabetic therapy: cinnamon (*Cinnamomum cassia*), dill (*Anethum graveolens)*, European blueberry (*Vaccinium myrtillus*), nettle (*Urtica dioica*), gurmar (*Gymnema sylvestre*), Tea “György” (fantasy name; contains dandelion-*Taraxacum officinale*, nettle-*Urticadioica*, perforate St. John’s wort-*Hypericum perforatum*, European blueberry-*Vaccinium myrtillus*, and chicory-*Cichorium intybus*) by three patients.

Patients have not used homeopathic remedies.

Reported CAM therapies alongside their ADRs and potential drug–herb interactions are listed in Table 1. Among ADRs are gastrointestinal (GI) and central nervous system (CNS) symptoms and dermatological conditions. In drug–herb interactions, a wide range of medicines are affected due to the modulation of CYP enzymes such as CYP3A4 and CYP2C9, which are responsible for the metabolism of many medicines.

**TABLE 1:** Reported complementary and alternative therapies (CAM) and their possible consequences.

Name of CAM	Is it used to treat epilepsy or diabetes mellitus?	Adverse drug reaction	Drug–Herbal interaction
Lemon balm (*Melissa officinalis*)	Yes (both hypoglycemic and anticonvulsant effects) (Chung et al., 2010; Ghayour et al., 2012)	Sleep disturbances and tiredness may happen (Cerny and Schmid, 1999)	It may interact with: Thyroid medications
			Barbiturates
			Sedatives
			Nicotine and scopolamine
			SSRIs (Posadzki et al., 2013; Holcomb, 2021)
Pomegranate (*Punica granatum*)	Yes (both hypoglycemic and anticonvulsant effects) (Banihani et al., 2013; Gollapalle et al., 2021)	No ADR in case of moderate consumption (Viladomiu et al., 2013)	Inhibits CYP3A4 and CYP2C9, so increases levels of drugs that are substrates of them (Andrade, 2014)
Valerian (*Valeriana officinalis*)	Yes (hypoglycemic effects and traditionally used in epilepsy) (Wang and Ng, 1999; Pilerood and Prakash, 2013)	Rare adverse events	*In vitro* evidence demonstrates CYP3A4 inhibition by valerian and thus the potential to interact with CYP3A4 substrates (e.g., atorvastatin and warfarin) (Cramer et al., 2006)
		-Dizziness	
		-Migraine	
		-GIT effects (Hadley and Petry, 2003)	
Rose hip (*Rosa canina*)	Traditional folk remedy for diabetes (Patel, 2017)	Gastrointestinal discomfort (Christensen et al., 2008)	Marginal effect on CYP3A4 activity (Kikuchi et al., 2017)
*Aloe vera*	Yes (both hypoglycemic and anticonvulsant effects) (Choudhary et al., 2011; Rathor et al., 2014)	-Carcinogenic activity in rats (Group 2B human carcinogen)	-Reduction in prostaglandin synthesis, which may inhibit secondary aggregation of platelets
		-Skin irritation	-The increased loss of potassium may potentiate the actions of conventional drugs, such as cardiac glycosides and corticosteroids (Boudreau and Beland, 2006)
		-Hives	
		-Cramping and diarrhea	
		-Hepatotoxicity (Guo and Mei, 2016)	
Sedacur contains:	-Look above for Lemon balm and Valerian	- Look above for Lemon balm and Valerian	- Look above for Lemon balm and Valerian
-Lemon balm			-Hop interacts with both serotonin (5-HT6) and melatonin (ML1) receptor subtypes in the CNS
- Valerian	- Hop (*Humulus lupulus*) has shown both hypoglycemic and anticonvulsant properties (Lee et al., 1993; Pavel et al., 2017)	-No significant adverse effects were reported from hop (Kyrou et al., 2017)	
-Hop (*Humulus lupulus*)			-Hop can inhibit CYP enzymes (Yuan et al., 2014)
Linden (*Tilia*)	Anticonvulsant effect (Cárdenas et al., 2014)	No reported adverse effects in literature apart from rare reports about allergic reactions (De Smet et al., 1993)	No documented drug interactions have been reported (Bédard, 2004)
Mint (*Mentha*)	Yes (both hypoglycemic and anticonvulsant effects) (Tolstikova et al., 2009; Bayani et al., 2017)	It can be an irritant and may cause, although rarely, hypersensitivity reactions and provoke contact dermatitis (Spirling and Daniels, 2001)	-Increases topical absorption of 5-fluorouracil
			-Synergy effect with some antibacterials
			-Calcium channel-blocking activity, so it can have additive effect with antihypertensives
			-It may inhibit CYP3A4 (Keifer et al., 2008)
Thymus	Yes (both hypoglycemic and anticonvulsant effects) (Alamgeer Muhammad et al., 2013; Skalicka-Woźniak et al., 2018)	-Dermatologic or allergic reactions	-Decreased levels of thyroid hormones
			-*In vivo* estrogen and progesterone activity -Enhanced percutaneous absorption of 5-fluorouracil (Basch et al., 2004)
		-Conjunctivitis	
		- Pulmonary adverse effects like occupational asthma, alveolitis, and rhinitis	
		-Gastrointestinal adverse effects like heartburn, vomiting, diarrhea, and nausea	
		- Musculoskeletal adverse effects (Basch et al., 2004)	
Senna	Yes (both hypoglycemic and anticonvulsant effects) (Tochukwu et al., 2021)	-Mild abdominal complaints like cramps or abdominal pain	-It can decrease absorption of other drugs (Fugh-Berman, 2000)
		-Discoloration of urine	
		-Hemorrhoid congestion	
		-Diarrhea and loss of electrolytes	
		in case of prolonged use or overdosing (Mascolo et al., 1998)	
Cinnamon (*Cinnamomum cassia*)	Yes (both hypoglycemic and anticonvulsant effects) (Lu et al., 2011; Belemkar et al., 2013)	Despite being safe as spice or flavoring agent, significant adverse effects occurred at larger doses or longer use duration	Interaction with CYP2A6 (Espiritu et al., 2020)
		-The most frequent adverse events were gastrointestinal disorders and allergic reactions (Hajimonfarednejad et al., 2019)	
Dill (*Anethum graveolens*)	Yes (both hypoglycemic and anticonvulsant effects) (Arash et al., 2013; Goodarzi et al., 2016)	-Skin irritation	Interaction with antidiabetic drugs because of cardiovascular effects (Cameron et al., 2012)
		-It can reduce the duration of labor (Talebi et al., 2020).	
European blueberry (*Vaccinium myrtillus*)	Yes (both hypoglycemic and anticonvulsant effects) (Collins et al., 2006; Grace et al., 2009)	-Gastrointestinal discomfort	Mild modulation of CYP enzymes (Prokop et al., 2019)
		-Drop in blood pressure	
		-Bleeding risk (Ulbricht et al., 2009)	
Nettle (*Urtica dioica*)	Yes (both hypoglycemic and anticonvulsant effects) (Kavalali et al., 2003; Loshali et al., 2021)	-Urticarial rash	-Increased sensitivity of breast cancer cells to paclitaxel
		-Upset stomach (Setty and Sigal, 2005)	- Nettle seed extract may have the potential to inhibit and/or induce the metabolism of certain coadministered drugs due to effects on enzymes like CYP2C6 and CYP2E1 (Agus et al., 2009; Mohammadi et al., 2016)
Gurmar (*Gymnema sylvestre*)	Yes (both hypoglycemic and anticonvulsant effects) (Murakami et al., 1996; Dwivedi and Gupta, 2017)	No clinically significant adverse effect (Ulbricht et al., 2011)	-Potentiating the effect of hypoglycemic drugs
			-Reduction of serum triglycerides, total cholesterol, VLDL, and LDL, which can potentiate the effects of antihyperlipidemic medications (Ulbricht et al., 2011)
Tea “György” contains:	-Look above for Nettle and European blueberry	-Look above for Nettle and European blueberry	- Look above for Nettle and European blueberry
			- Dandelion can potentiate the effects of other antihyperlipidemics (Sweeney et al., 2005)
- Dandelion (*Taraxacum officinale*)	-Dandelion has hypoglycemic effects (Li et al., 2021)	- Adverse effects of Dandelion include stomach discomfort, diarrhea, and heartburn (Sweeney et al., 2005)	
			- Chicory may induce CYP enzymes (Rasmussen et al., 2011)
			- St. John’s wort induces cytochrome P450 isoenzymes such as CYP3A4, CYP2C9, CYP1A2, and the transport protein P-glycoprotein causing clinically significant interactions with prescribed medicines including warfarin, phenprocoumon, cyclosporin, HIV protease inhibitors, theophylline, digoxin, and oral contraceptives resulting in a decrease in concentration or effect of the medicines (Henderson et al., 2002)
- Nettle (*Urtica dioica*)	-Chicory has hypoglycemic effects (Draz et al., 2010)		
		-Chicory is generally regarded as safe (Perović et al., 2021)	
-perforate St. John’s wort (*Hypericum perforatum*)	- St. John’s wort has both anticonvulsant effects and hypoglycemic effects (Hosseinzadeh et al., 2005; Arokiyaraj et al., 2011)		
		-Adverse effects of St. John’s wort include gastrointestinal symptoms, dizziness, confusion, tiredness, sedation, and dry mouth (Henderson et al., 2002)	
-European blueberry (*Vaccinium myrtillus*)			
-Chicory (*Cichorium intybus*)			

Basic patients’ characteristics were assessed in Table 2. Comparing patients with epilepsy and DM, statistically significant differences were revealed in body weight, smoking status, and alcohol consumption.

**TABLE 2:** Basic characteristics of overall study population, patients living with epilepsy, and patients living with diabetes mellitus.

	All patients (*N* = 227)	Patients with epilepsy (*N* = 127)	Patients with diabetes mellitus (*N* = 100)	*p*-Value
Mean body weight (kg ± SD)	80.3 ± 17.3	76.1 ± 17.8	85.7 ± 15	0.0089
Mean age (year ± SD)	54.54 ± 17.33	45.1 ± 16.1	65.51 ± 8.66	**<0.00001**
**Education**				0.65
*Primary school*	64	28	36	
*High school*	111	66	45	
*University*	44	28	16	
*Doctoral school*	1	1	0	
**Smoking status**				**<0.00001**
*Smoker*	30	22	8	
*Never smoked*	121	80	41	
*Stopped smoking*	71	23	48	
Physical activity	Yes: 190 No: 31	103 20	87 11	0.28
Alcohol consumption	No: 179 Yes: 42	113 12	66 30	**0.000048**

*Bold values mean statistically significant differences between the epilepsy and diabetes group if p < 0.05.*

Patients living with epilepsy had better disease control (73.2% vs. 28%), but CAM was not significant among the groups despite that 7% of the patients with epilepsy had controlled disease using CAM vs. 1% of patients with DM. Adherence rate was higher among patients with epilepsy (Table 3).

**TABLE 3:** Disease and treatment characteristics.

	All patients (*N* = 227)	Patients with epilepsy (*N* = 127)	Patients with diabetes mellitus (*N* = 100)	*p*-Value
Monotherapy	112	73	39	0.14
Bitherapy	52	27	25	
Polytherapy	32	12	20	
Not stated	31	15	16	
**Controlled disease**	121	93	28	**<0.00001**
*noCAM use*	*111*	*84*	*27*	0.45
*CAM use*	*10*	*9*	*1*	
Adverse drug reactions from prescribed medicines	No: 177	No: 101	No: 76	0.24
	Yes: 26	Yes: 18	Yes: 8	
Adherence to prescribed medicines	Always: 197	Always: 114	Always: 83	**0.047**
	Less often: 5	Less often: 3	Less often: 2	
	Poor: 6	Poor: 0	Poor: 6	
Satisfaction with prescribed medicines	Yes: 197	Yes: 118	Yes: 79	0.12
	No: 11	No: 4	No: 7	
Quality of life assessment (Likert scale)				0.74
***1***	106	77	29	
***2***	79	31	48	
***3***	4	2	2	
***4***	1	0	1	
***5***	0	0	0	

*Bold values mean statistically significant differences between the epilepsy and diabetes group if p < 0.05.*

The ratio of CAM use was 9.7% in the overall study population. Among patients with epilepsy, it was less than that among diabetic patients; 7.9 and 12%, respectively. Interestingly, among patients living with epilepsy, CAM users were mainly younger, while the CAM-using DM patients were members of the elderly population. Only two patients (9.1% of CAM users–prevalence of CAM ADR) reported ADR from the used CAM (Table 4).

**TABLE 4:** Complementary and alternative medicine (CAM) usage-related issues.

	All patients (*N* = 227)	Patients with epilepsy (*N* = 127)	Patients with diabetes mellitus (*N* = 100)	*p*-Value
CAM users	22	10	12	0.3
**By age group**	–	–	–	0.2
≤*65 years old*	*11*	*7*	*4*	
>*65 years old*	*11*	*3*	*8*	
CAM ADR	2	0	2	

*Significant difference between the epilepsy and diabetes group was if p < 0.05.*

Nevertheless, we failed to find a significant association between CAM use and age, gender, adherence to prescribed medicine (although it should be noted that at 90% CI, it is significant), ADR due to prescribed medicine, control of disease, smoking, satisfaction with prescribed medicines, and education. A patient reporting alcohol consumption and physical activity had 4.56 and 9.1 times odds of using CAM, respectively, compared to a person who did not (Table 5).

**TABLE 5:** Factors possibly affecting the use of complementary and alternative medicine (CAM).

	CAM user (*N* = 22)	Non-CAM user (*N* = 205)	Odds ratio (95% CI; *p*-Value)
Age > 65 years	10	51	2.02 (0.83–5.02; 0.12)
Gender male	12	90	1.17 (0.48–2.85; 0.72)
Obesity	6	16	0.7 (0.26–1.88; 0.49)
Epilepsy	10	108	0.54 (0.22–1.31, 0.18)
Adherence to prescribed medicine	22	159	13.12 (0.78–220.41; 0.0096)
ADR	4	20	0.56 (0.17–1.85; 0.35)
**Control of the disease**	10	97	0.93 (0.38–2.24; 1.0)
*Epilepsy*	*9*	*84*	*3.54 (0.43–29.01; 0.29)*
*Diabetes mellitus*	*1*	*13*	*0.71 (0.08–6.9; 1.0)*
**Smoking**			
*Yes*	2	23	0.79 (0.17–3.61; 1.0)
*Never*	10	95	0.96 (0.4–2.33; 1.0)
*Stopped*	10	56	2.22 (0.91–5.42; 0.86)
Alcohol consumption	9	27	**4.56 (1.78–11.7; 0.0026)**
Physical activity	21	143	**9.1 (1.2–69.19; 0.01)**
*Satisfaction*	19	160	0.28 (0.07–1.16; 0.58)
**Education**			
*Primary school*	4	49	0.56 (0.18–1.75; 0.32)
*High school*	13	88	1.41 (0.57–3.47; 0.45)
*University*	5	36	1.13 (0.39–3.26; 0.82)
*Doctoral school*	0	1	N/A

*Bold values mean statistically significant differences.*

## Discussion

In this study, we have included two chronic diseases requiring lifelong treatment. They should be given special attention not only by the patients but by the relatives as well. Epileptic seizure is unforeseen and, in many cases, dramatic. Besides this, epilepsy is stigmatizing and enigmatic, which may motivate patients to use uncommon treatments. These might even give the patient an illusion that they can rule their whimsical disease by themselves. It is a well-known fact that the consequences of DM can be reduced with diet and changes in lifestyle, and this depends on the patients. The increased interest in CAM and potential source of ADR or interaction play a pivotal role in having information on the use of CAM in order to find the best-tailored therapy for each patient.

### Characteristics of Complementary and Alternative Medicine Use

A systematic review revealed the prevalence of use of any CAM between 9.8 and 76% in the general population and concluded that periodic surveys were important to monitor CAM use at the population level (Harris et al., 2012).

In our study, the overall prevalence was similar to the findings of the above systematic review, but fewer users (7.9%) could be detected among patients with epilepsy. By comparing two groups with a similar cultural background, we could decrease the bias and assume that the characteristics of the diseases contribute to differences in findings (Barner et al., 2010). If compared to the population with DM, treatment characteristics differed in some points such as adherence, which could be attributed to the unexpectedness and dramatic nature of epilepsy; in contrast, in DM, high blood sugar level is “just” a laboratory finding. A seizure is an unpleasant feedback that might affect the quality of life (e.g., job, driving license, and relationships), and most patients try to avoid it. Epileptologists believe that continuous care and a good physician–patient relationship improve trust and sincerity.

### Risk Factors of Complementary and Alternative Medicine Use

Unlike in the article by Nahin et al. (2007), smoking and obesity were not confirmed as risk factors related to CAM use in the current study population. Physical activity was linked to CAM use, which may suggest health-conscious people may want to rule their disease by using additional treatment and make every effort for their wellbeing. This hypothesis is supported by other findings in this study, for instance, CAM use was independent of the control of disease, satisfaction with prescribed medicines, and quality of life assessment.

Surprisingly, alcohol consumption elevated reliance on using CAM by more than four times. But patients have not reported heavy drinking; only one patient confessed daily drinking of two bottles of beer. In a Norwegian study among women, similarly to our findings, more frequent natural medicine use was detected among frequent alcohol drinkers (Sivertsen et al., 2018).

Complementary and alternative medicine users were younger in the “epileptic” group, but it can be attributed to their younger mean age than patients with DM.

### Adverse Drug Reaction

In Table 1, most CAMs are used for both epilepsy and DM patients. Despite the small size of the study population, 9.1% of CAM users reported ADRs. These CAMs typically modulated CYP enzymes. The CAMs used by our patients might have had interactions with AEDs, partially on CYP enzymes, partially that absorption could have been disturbed. Being aware of their use, it might prevent an unnecessary change in AED therapy. This finding emphasizes the importance of history taking, which should include CAM use and, in addition to patients’ education, the knowledge of different CAM effects by the treating team including clinical pharmacists.

## Conclusion

It must be mentioned that although patients using CAM are only a minority in the studied population, attention should be paid to reliance on CAM during the follow-up. Our finding that health-conscious patients tend to use CAM more often (than the general population) may indicate it is necessary to discuss CAM usage honestly. CAMs capable of modulating CYP enzymes were the most popular among users, but they led to interactions with medication and resulted in ADRs. This shows that educating patients and treating them by a team including a clinical pharmacist in this field are essential.

## Limitations

The authors are aware that the study has several/certain limitations. Nevertheless, the strength of the study is the cross-sectional view and honest responses due to anonymously collected questionnaires. Although the size of the sample was limited, only patients willing to participate were included in the study, which resulted in a selection bias. The facts mentioned above might imply that the study was underpowered to identify all clinically important predictors of CAM use.

## Data Availability Statement

The raw data supporting the conclusions of this article will be made available by the authors, without undue reservation.

## Ethics Statement

The studies involving human participants were reviewed and approved by Institutional Ethics Committee of the University of Debrecen (Registration Number: DE RKEB/IKEB 5098-2018 and 5099-2018). Written informed consent for participation was not required for this study in accordance with the national legislation and the institutional requirements.

## Author Contributions

All authors listed have made a substantial, direct, and intellectual contribution to the work, and approved it for publication.

## Conflict of Interest

The authors declare that the research was conducted in the absence of any commercial or financial relationships that could be construed as a potential conflict of interest.

## Publisher’s Note

All claims expressed in this article are solely those of the authors and do not necessarily represent those of their affiliated organizations, or those of the publisher, the editors and the reviewers. Any product that may be evaluated in this article, or claim that may be made by its manufacturer, is not guaranteed or endorsed by the publisher.
